# Lentinan protects pancreatic β cells from STZ‐induced damage

**DOI:** 10.1111/jcmm.12865

**Published:** 2016-07-22

**Authors:** Yaqin Zhang, Hongliang Mei, Wei Shan, Li Shi, Xiaoai Chang, Yunxia Zhu, Fang Chen, Xiao Han

**Affiliations:** ^1^ Department of Biochemistry and Molecular BiologyKey Laboratory of Human Functional Genomics of Jiangsu ProvinceNanjing Medical UniversityNanjingJiangsuChina; ^2^ Department of PharmacologyCollege of MedicineNanjing Medical UniversityNanjingJiangsuChina; ^3^ The Affiliated Drum Tower Hospital of Nanjing Medical UniversityNanjingJiangsuChina; ^4^ Changzhou No.2 People's HospitalThe Affiliated Hospital of Nanjing Medical UniversityChangzhouJiangsuChina

**Keywords:** lentinan, pancreatic β cell, apoptosis, dysfunction, antioxidant activity

## Abstract

Pancreatic β‐cell death or dysfunction mediated by oxidative stress underlies the development and progression of diabetes mellitus (DM). In this study, we evaluated the effect of lentinan (LNT), an active ingredient purified from the bodies of *Lentinus edodes*, on pancreatic β‐cell apoptosis and dysfunction caused by streptozotocin (STZ) and the possible mechanisms implicated. The rat insulinoma cell line INS‐1 were pre‐treated with the indicated concentration of LNT for 30 min. and then incubated for 24 hrs with or without 0.5 mM STZ. We found that STZ treatment causes apoptosis of INS‐1 cells by enhancement of intracellular reactive oxygen species (ROS) accumulation, inducible nitric oxide synthase (iNOS) expression and nitric oxide release and activation of the c‐jun N‐terminal kinase (JNK) and p38 mitogen‐activated protein kinase (MAPK) signalling pathways. However, LNT significantly increased cell viability and effectively attenuated STZ‐induced ROS production, iNOS expression and nitric oxide release and the activation of JNK and p38 MAPK in a dose‐dependent manner *in vitro*. Moreover, LNT dose‐dependently prevented STZ‐induced inhibition of insulin synthesis by blocking the activation of nuclear factor kappa beta and increasing the level of Pdx‐1 in INS‐1 cells. Together these findings suggest that LNT could protect against pancreatic β‐cell apoptosis and dysfunction caused by STZ and therefore may be a potential pharmacological agent for preventing pancreatic β‐cell damage caused by oxidative stress associated with diabetes.

## Introduction

Diabetes mellitus (DM) has become an epidemic and now represents a major health problem worldwide 1, 2. It is commonly known that β‐cell death or dysfunction plays a critical role in the pathophysiological progression of both type 1 and type 2 diabetes 3, 4, 5, 6, 7, 8, 9. Although the main cause is not yet clear, glucolopotoxicity, cytokines and genetic factors are known to induce β‐cell apoptosis by oxidative stress in diabetic subjects and rodents 10, 11, 12. Importantly, oxidative stress is also associated with long‐term damage, dysfunction and eventually the failure of organs, especially the eyes, heart, kidneys, nerves and vascular system 13, 14, 15. Therefore, antioxidant agents could be helpful in reducing oxidative stress, and thus in preventing or slowing down the process of β‐cell death or dysfunction in the onset and development of DM.

Lentinan (LNT), a β‐1,3 β‐glucan with β‐1,6 branches, is an active ingredient purified from the bodies of *Lentinus edodes* and has been used in traditional medicine 16, 17. Many previous studies have demonstrated that LNT exhibited multiple bioactivities, including antioxidation 18, antitumour 19, antiviral 20, antibacterial 21, 22, anti‐inflammation 23 and immunoregulation 24. However, LNT has not been used for the treatment of diabetes, and an effect of LNT on β cells has not been reported. Therefore, in this study we designed experiments to investigate whether LNT can protect against pancreatic β‐cell apoptosis and dysfunction induced by streptozotocin (STZ). Furthermore, we investigate the mechanisms underlying of this protective action, to determine whether it might be a potential pharmacological treatment of stress‐mediated diabetes.

## Materials and methods

### Cell culture

A rat INS‐1 cell line, purchased from American Type Culture Collection (ATCC, Manassas, VA, USA), retains physiological characteristics of normal β cells. INS‐1 cells (passages 10–20) were grown in RPMI 1640 medium (Hyclone, Logan, UT, USA), containing 6% fetal bovine serum (FBS) (vol./vol.), 50 μmol/l β ‐mercaptoethanol, 1 mmol/l sodium pyruvate, 2 mmol/l L‐glutamine, 100 U/ml penicillin, 100 μg/ml streptomycin (all from Sigma‐Aldrich, St. Louis, MO, USA) and cultured at 37°C in a humidified atmosphere containing 95% air and 5% CO_2_.

### Cell viability assay

Cell viability was determined by an MTT [3‐(4,5‐dimethyl‐2‐thiazolyl)‐2,5‐diphenyl‐2‐H‐tetrazolium bromide] assay. Briefly, INS‐1 cells were seeded in 96‐well plates at a density of 1 × 10^4^ cells per well. Some cells were treated with STZ at concentrations of 0, 0.25, 0.5, 1 and 2 mmol/l for 24 hrs, followed by incubation with MTT (0.5 mg/ml, Sigma‐Aldrich) for 4 hrs. Other cells were treated with LNT (Sigma‐Aldrich), which was dissolved in physiological saline. Following pre‐incubation with LNT at concentrations of 0, 50, 100, 200 and 400 μg/ml for 30 min., the cells were exposed to STZ (0.5 mmol/l) and LNT (0, 50, 100, 200 and 400 μg/ml) for an additional 24 hrs. Each well was then supplemented with 10 μl MTT and incubated for 4 hrs at 37°C. Then, the formazan precipitate was dissolved in dimethyl‐sulfoxide (Sigma‐Aldrich) and the absorbance at 490 or 570 nm was determined with a microplate reader (Perlong, Beijing, China).

### EdU proliferation assay

Cell proliferation was measured by 5‐ethynyl‐2′‐deoxyuridine (EdU) assay using an EdU assay kit (Ribobio, Guangzhou, China) according to the manufacturer's instructions. Briefly, INS‐1 cells were seeded at 2 × 10^3^ cells per well in 96‐well plates and pre‐incubated with indicated LNT (50, 100, 200 and 400 μg/ml) in a humidified atmosphere containing 5% CO_2_ at 37°C for 30 min. After 30 min. of incubation, the cells were treated with STZ (0.5 mM) and the indicated concentration of LNT and further incubated for 24 hrs. Then, the cells were incubated with 50 μM EdU for additional 3–4 hrs at 37°C before fixation and permeabilization. After 3× washes with PBS, the cell nuclei were stained with 100 μl of Hoechst 33342 (1 μg/ml) for 5–10 min. and visualized under a fluorescent microscope (Olympus, Tokyo, Japan).

### TUNEL staining assay

INS‐1 cells were cultured on coverglass in 12‐well plates. After 24 hrs treatment as described above, the apoptotic cells were stained in a terminal deoxynucleotidyl transferase mediated nick‐end labelling (TUNEL) assay according to the instruction of the kit manufacturer (In Situ Cell Death Detection Kit; Roche, Basel, Switzerland) 25. Apoptotic cells were stained by green fluorescence, and all cells were marked with blue fluorescence using Hoechst. The apoptotic ratio was calculated as tunnel‐positive cells divided by total cell number. The number of cells was counted in five random fields from three different slides at 400× magnification. An average for the percentage of tunnel‐positive cells was taken over these fields.

### Flow cytometry analysis

INS‐1 cells (1 × 10^6^ cells per well) were cultured in 6‐well plates and pre‐treated with LNT or anisomycin (Am; Sigma‐Aldrich), a direct activator of JNK and p38, for 30 min. and then exposed to STZ or LNT an additional 24 hrs. Thereafter, the cells were digested with 0.25% trypsin and incubated with 20 μl of binding buffer, 5 μl of Annexin V‐FITC and 5 μl of propidium iodide. After incubation at room temperature in the dark for 15 min., cells were analysed by flow cytometry 26. The results were calculated using the CellQuest^™^ Pro software (BD Biosciences, San Jose, CA, USA) and expressed as the percentage of apoptotic cells from the total cells.

### Measurement of the intracellular reactive oxygen species generation

A commercial Cellular Reactive Oxygen Species (ROS) Detection Assay Kit (Red Fluorescence; Abcam, Cambridge, UK) was used to measure intracellular ROS according to the instruction of the kit manufacturer. Briefly, INS‐1 cells (2 × 10^4^ cells/100 μl) were seeded in 96‐well black plates and pre‐incubated with indicated LNT (50, 100, 200 and 400 μg/ml) or Am in a humidified atmosphere containing 5% CO_2_ at 37°C for 30 min. After 30 min. of incubation, the cells were treated with STZ (0.5 mM) or/and the indicated concentration of LNT and further incubated for 24 hrs. Thereafter, the medium was removed, and the cells were washed twice with PBS, and incubated with 100 μl ROS red working solution at 37°C for 1–2 hrs. After incubation, the cells were washed and re‐suspended with ice‐cold PBS. Fluorescence was measured at Ex/Em = 520/605 nm using a fluorescence plate reader (BMG LABTECH GmbH, Offenburg, Germany).

### Nitric oxide assay

Nitric oxide concentration in the cell culture was measured using a microplate assay method, as previously described 27. Briefly, cells were seeded in 48‐well plates (2 × 10^4^ cells per well in 200 μl of medium) and treated for 30 min. at 37°C with LNT (0, 50, 100, 200 and 400 μg/ml). After 30 min. of incubation, 0.5 mM STZ was added, and the plate was incubated at 37°C for 24 hrs. The medium was sampled for nitric oxide determination using the Griess method. The absorbance at 540 nm was determined using a microplate reader. The nitric oxide content was determined using sodium nitrite as a standard. Each experiment was performed in triplicate and repeated three times independently for reproducibility.

### Real‐time RT‐PCR assay

INS‐1 cells were cultured and treated as described above. Total RNA was extracted using Trizol reagent (Invitrogen, Grand Island, NY, USA). First‐strand cDNA synthesis was performed with 1 μg of total RNA and an avian myeloblastosis virus reverse transcription system. The primers were designed using the software Primer Express (Applied Biosystems, Foster City, CA, USA). Real‐time quantitative PCR was performed with the SYBR Green PCR Master Mix and ABI Prism 7000 Sequence Detection System (Applied Biosystems). All the data were analysed using the expression of β‐actin as a reference.

### Western blot analysis

INS‐1 cells were plated into 6‐well plates at a density of 1 × 10^6^ cells/well and treated as described above. After exposure to STZ and LNT for 24 hrs, cells were rinsed twice with pre‐cooled PBS and lysed on ice with RIPA lysis buffer containing 1 mmol/l PMSF. Protein concentrations of the extracts were determined with a bicinchoninic acid protein assay kit (Thermo Scientific Pierce, Rockford, IL, USA), using bovine serum albumin as a standard (Beyotime Biotech, Haimen, China). Proteins (40–60 μg protein/lane) were separated by electrophoresis on 12% polyacrylamide gels, and the bands were subsequently transferred onto polyvinylidene fluoride membranes (Millipore, Billerica, MA, USA). Membranes were blocked in PBST/5% non‐fat dry milk powder and incubated with antibodies against inducible nitric oxide synthase (iNOS), NF‐κB p65, Histone H3, GAPDH (Santa Cruz Biotechnology, Santa Cruz, CA, USA), phospho‐c‐Jun N‐terminal kinase (P‐JNK), total JNK (T‐JNK), phospho‐p38 Map out (P‐P38), total p38 (T‐P38), phosphorylated ERK 1/2 (P‐ERK1/2) and total ERK1/2 (T‐ERK1/2) (Abcam). After washes by Tris‐buffered saline (TBS)‐Tween 20 (0.05%), a second antibody conjugated to HRP (Santa Cruz Biotechnology) was used to incubate the membranes at room temperature for 1 hr. Finally, membranes were developed with Super Signal West Pico chemiluminescence reagent (Thermo Scientific Pierce), then visualized on X‐ray films. ImageJ (National Institutes of Health, Bethesda, MD, USA) was used for quantification of immunoblots.

### Insulin content assay

INS‐1 cells were seeded in 24‐well plates and treated as described above. After 24 hrs, cells were washed twice with PBS (pH 7.4) at 0°C and extracted with acid/ethanol (0.15 M HCl in 75% ethanol in H_2_O) for 16 hrs at 0°C. Supernatants were collected and stored at −80°C until insulin determination was carried out by ELISA. The results were normalized to the total protein concentration 28.

### Statistical analysis

All the data are expressed as the mean ± S.E.M. of three independent experiments. Statistical significance was determined using Student's *t*‐test or a one‐way anova. A value of *P* < 0.05 was considered statistically significant.

## Results

### LNT protects INS‐1 cells from STZ‐induced apoptosis

Streptozotocin has been widely used as an inducer for apoptosis of β cells, which leads to β‐cell destruction and reduced insulin secretion 29. To identify the functional concentration of STZ in INS‐1 cells, a dose–response analysis was performed. As shown in Figure S1A, the MTT analysis clearly showed that STZ reduced cell viability in a dose‐dependent manner. Based on these results, 0.5 mM STZ was selected for subsequent experiments aiming to induce a mild apoptosis. To test whether the survival of cells was affected by LNT, INS‐1 cells were incubated with LNT (0–400 μg/ml) for 24 hrs and cell viability was determined in a MTT assay. As shown in Figure S1B, when the cells were treated with LNT alone, cell viability was not largely influenced by LNT concentrations, which suggests that LNT did not show significant cytotoxicity.

**Figure 1:**
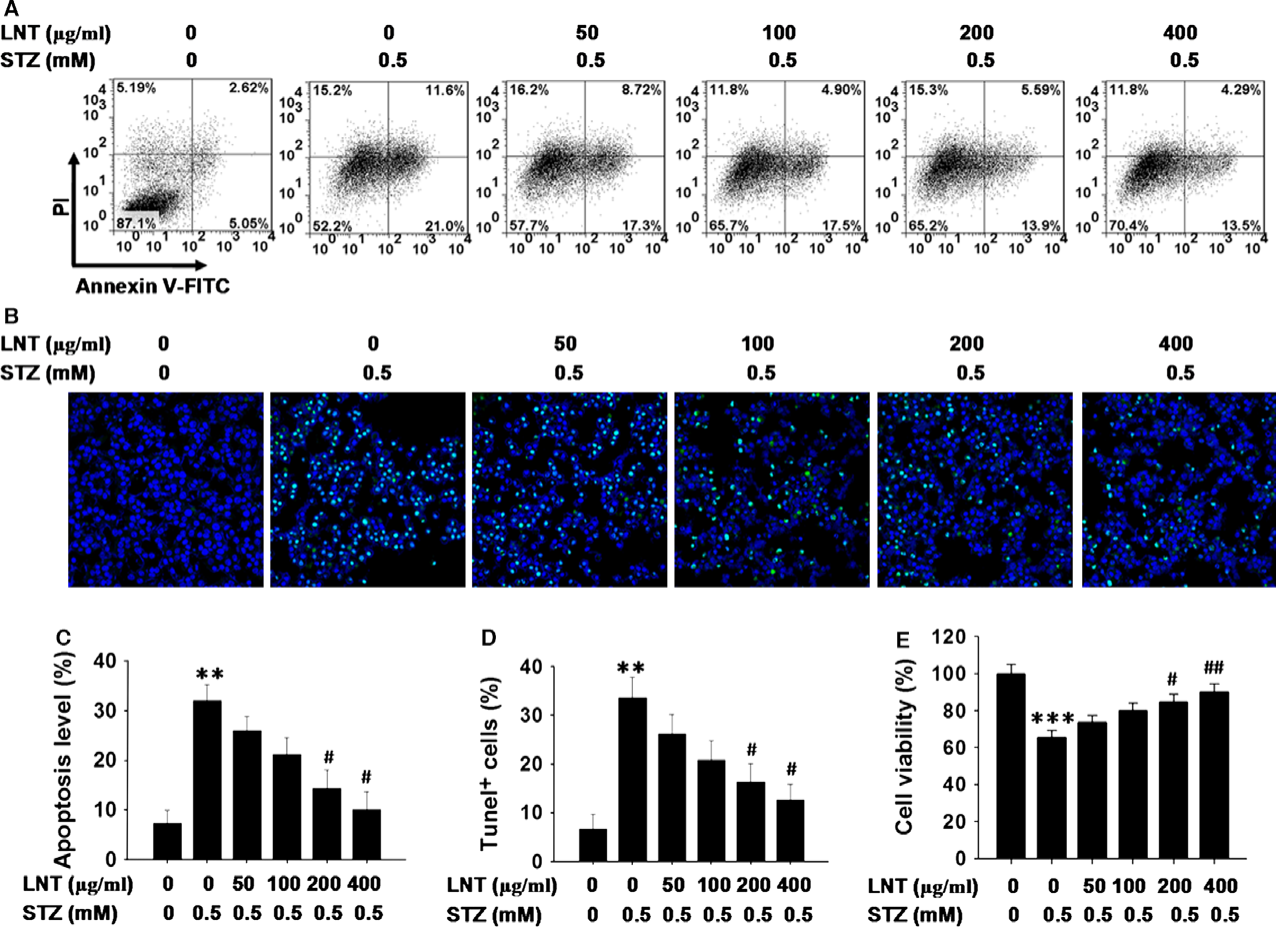
Effect of LNT on apoptosis of INS‐1 cells exposed to STZ. (**A**) Cells were pre‐treated with the indicated concentration of LNT for 30 min. and then incubated for 24 hrs with or without 0.5 mM STZ. Cell apoptosis was detected using flow cytometric assay with Annexin V‐FITC and PI‐staining. (**B**) TUNEL staining of cellular apoptosis of INS‐1 cells treated as in **A**. The images of TUNEL positive cells were captured with a confocal laser scanning microscopy (400× magnification). (**C**) Quantitative analysis of cellular apoptosis detected by flow cytometric measurements. (**D**) Quantitative analysis of TUNEL assay results. (**E**) Cell viability was further evaluated in INS‐1 cells treated as in **A** using an MTT assay. All the results are presented as the means ± S.E.M. for three independent experiments. **P < 0.01, ***P < 0.001 compared to the untreated control group; ^#^ *P* < 0.05, ^##^ *P* < 0.01 compared to the STZ group.

To further investigate the potential role of LNT in INS‐1 apoptosis‐induced by STZ, we pre‐treated INS‐1 cells with various concentrations of LNT for 30 min., then cultured the cells with or without STZ for an additional 24 hrs and analysed the apoptosis and survival levels of the treated cells. As depicted in Figure 1A and C, a flow cytometric assay demonstrated that exposure to STZ (0.5 mM) markedly increased the apoptotic rate of cells, which was consistent with the MTT assay results (Fig. S1A). In contrast, cells co‐cultured with 200 and 400 μg/ml of LNT for 24 hrs showed a significant reduction in cell apoptosis. In addition, we observed a similar inhibitory effect of LNT on STZ‐induced apoptosis in INS‐1 cells using TUNEL (Fig. 1B and D). Furthermore, we also found that LNT can significantly improve cell viability (Fig. 1E) and proliferation (Fig. S1C) in a dose‐dependent manner in STZ‐treated INS‐1 cells. Collectively, these results suggested that LNT could protect INS‐1 cells from STZ‐induced apoptosis.

### LNT attenuates STZ‐induced ROS generation, nitric oxide production and iNOS expression in INS‐1 cells

**Figure 2:**
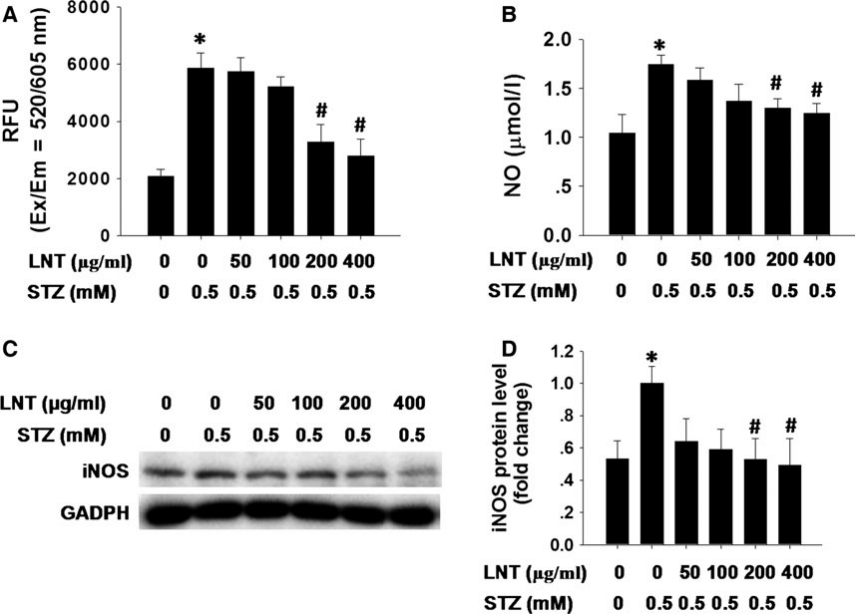
Effect of LNT on STZ‐induced ROS accumulation, nitric oxide synthesis and iNOS expression in INS‐1 cells. (**A**) Following pre‐incubation with LNT for 30 min., INS‐1 cells were treated with the indicated concentrations of LNT and STZ (0.5 mM) for 24 hrs. Intracellular ROS measured with Cellular Reactive Oxygen Species Detection Assay Kit (Red Fluorescence). The fluorescence signal was monitored at Ex/Em = 520/605 nm (cut‐off = 590 nm) with bottom read mode. (**B**) Cells were treated with different concentrations of LNT in the presence or absence of 0.5 mM STZ for 24 hrs. Nitric oxide production was then detected. (**C** and **D**) INS‐1 cells were treated as in **B**, and the protein level of iNOS was determined by Western blot analysis. *P < 0.05 compared to the untreated control group; ^#^ *P* < 0.05 compared to the STZ group.

Previous studies have shown that β‐cell toxicity of STZ is mediated by diverse mechanisms, including increased oxidative stress because of ROS production and nitric oxide release 30, 31, 32. We next investigated the effects of LNT on oxidative stress‐associated markers during STZ‐induced β‐cell apoptosis. As shown in Figure 2A, ROS generation was significantly increased in the STZ‐treated cells compared to the control group. In contrast, this elevation in ROS was markedly inhibited by LNT treatment in a dose‐dependent manner. Furthermore, 0.5 mM STZ treatment increased the synthesis of nitric oxide compared to nitric oxide synthesis measured in the control group. After treatment with LNT, total nitric oxide synthesis of INS‐1 cells treated with STZ was significantly inhibited in comparison to the cells treated with STZ only (Fig. 2B). Inducible nitric oxide synthase expression plays a critical role in sustaining elevation of nitric oxide, which was a major contributor in β‐cell dysfunction and apoptosis. Thereafter, to determine the effect of LNT on STZ‐induced nitric oxide production *via* up‐regulation of iNOS expression, we investigated the protein level of iNOS in a Western blot. Compared to normal cultured cells, STZ significantly increased iNOS expression. However, the addition of 200 and 400 μg/ml LNT to culture medium could effectively suppress the increased expression level of iNOS induced by STZ (Fig. 2C and D). Collectively, these findings suggest that LNT blocks STZ‐induced oxidative stress by reducing ROS levels, nitric oxide release and iNOS expression in INS‐1 cells.

### LNT inhibited JNK and p38 MAPK activation in STZ‐treated INS‐1 cells

**Figure 3:**
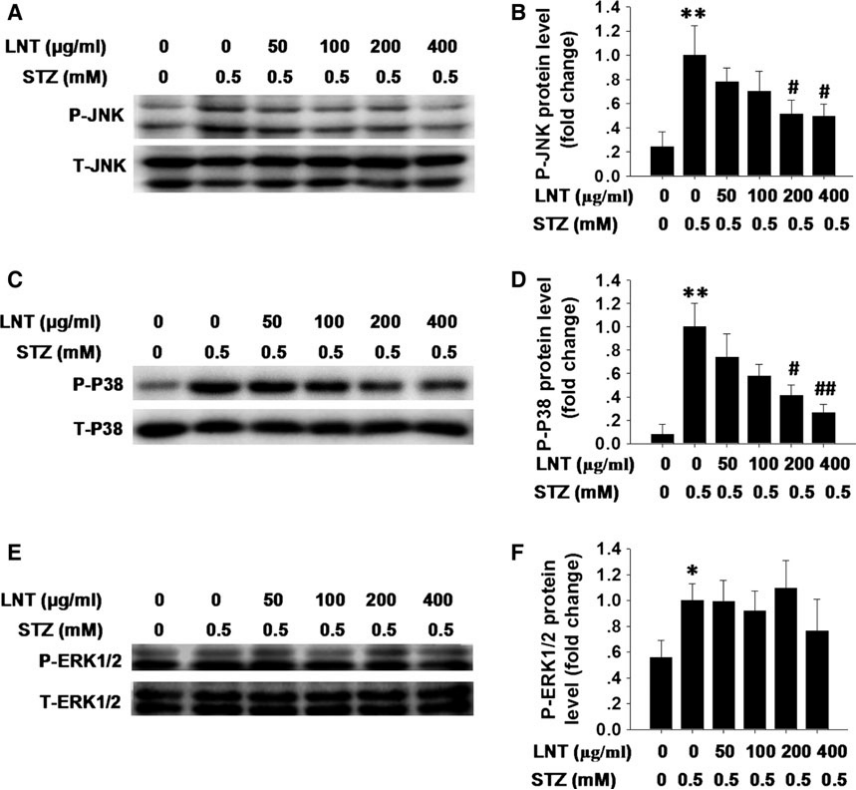
Effect of LNT on STZ‐induced JNK and p38 MAPK activation in INS‐1 cells. (**A**) Following pre‐incubation with LNT for 30 min., the cells were treated with the indicated concentrations of LNT and STZ (0.5 mM) for 24 hrs. Phospho (P‐JNK) and total (T‐JNK) form of JNK were analysed by Western blot assay. (**B**) Quantitative analysis of phosphorylation of JNK was detected as in **A**. (**C**) INS‐1 cells were treated as in **A**, and the phosphorylation of p38 MAPK (P‐P38) was determined by Western blot analysis. (**D**) Quantitative analysis of P‐P38 was detected as in **C**. (**E**) INS‐1 cells were treated as in **A**, and the phosphorylation of ERK1/2 (PERK1/2) was determined by Western blot analysis. (**F**) Quantitative analysis of phosphorylation of ERK1/2 was detected as in **E**. The data are presented as the means ±S.E.M. for three independent experiments. *P < 0.05, **P < 0.01 compared to the untreated control group; ^#^ *P* < 0.05, ^##^ *P* < 0.01 compared to the STZ group.

**Figure 4:**
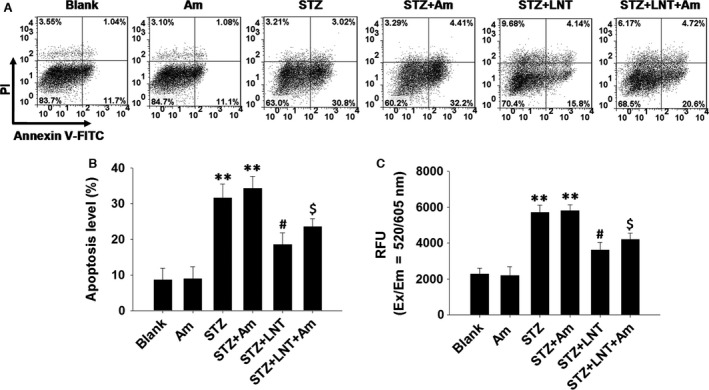
Possible mechanism for the effects of LNT on STZ‐induced apoptosis and ROS production. (**A**) After pre‐treatment with LNT (200 μg/ml) or anisomycin (Am, 1 μM) for 30 min., INS‐1 cells were treated with the LNT (200 μg/ml) and STZ (0.5 mM) for 24 hrs. Cell apoptosis was detected using flow cytometric assay with Annexin V‐FITC and PI‐staining. (**B**) Quantitative analysis of cellular apoptosis detected by flow cytometric measurements. (**C**) INS‐1 cells were treated as in **A**. ROS production was analysed using Cellular Reactive Oxygen Species Detection Assay Kit (Red Fluorescence). The fluorescence signal was monitored at Ex/Em = 520/605 nm (cut‐off = 590 nm) with bottom read mode. **P < 0.01 compared to the untreated control group; ^#^ *P* < 0.05 compared to the STZ group; ^$^ *P* < 0.05 compared to the STZ+Am group.

Reactive oxygen species‐induced oxidative stress plays a critical role in activating members of the mitogen‐activated protein kinase (MAPK) family, especially JNK and p38 MAPKs, to induce cell apoptosis 33. Given the results shown in Figure 2, we next detected the activation of JNK, p38 and ERK1/2 pathways in INS‐1 cells treated with LNT and with or without STZ. We found that STZ‐induced phosphorylation of JNK (Fig. 3A and B) and p38 MAPK (Fig. 3C and D) was significantly reduced by LNT treatment in a dose‐dependent manner. However, there was no obvious effect of LNT on STZ‐activated ERK1/2 phosphorylation (Fig. 3E and F). To further identify the potential mechanisms underlying the observed effects of LNT on STZ‐induced apoptosis and ROS production in INS‐1 cells, Am, a direct activator of JNK and p38 MAPK signalling pathways 34, was used. We found that a 24 hrs stimulation of INS‐1 cells with 1 μM Am did not affect cells viability, as assessed by the MTT assay (Fig. S1D). Subsequent co‐treatment with STZ and Am (1 μM) resulted in a significant elevation in the level of apoptosis (Fig. 4A and B, lane 4) and ROS generation (Fig. 4C, lane 4) in INS‐1 cells compared to untreated control. In contrast, this elevation in apoptotic cells (Fig. 4A and B, lane 6) and ROS (Fig. 4C, lane 6) were markedly inhibited by LNT treatment. Collectively, our results indicate that LNT protects INS‐1 cells against STZ‐induced apoptosis and oxidative stress through suppressing the JNK and p38 MAPK signalling pathway.

### LNT protects INS‐1 cells against STZ‐induced impairment of insulin synthesis

**Figure 5:**
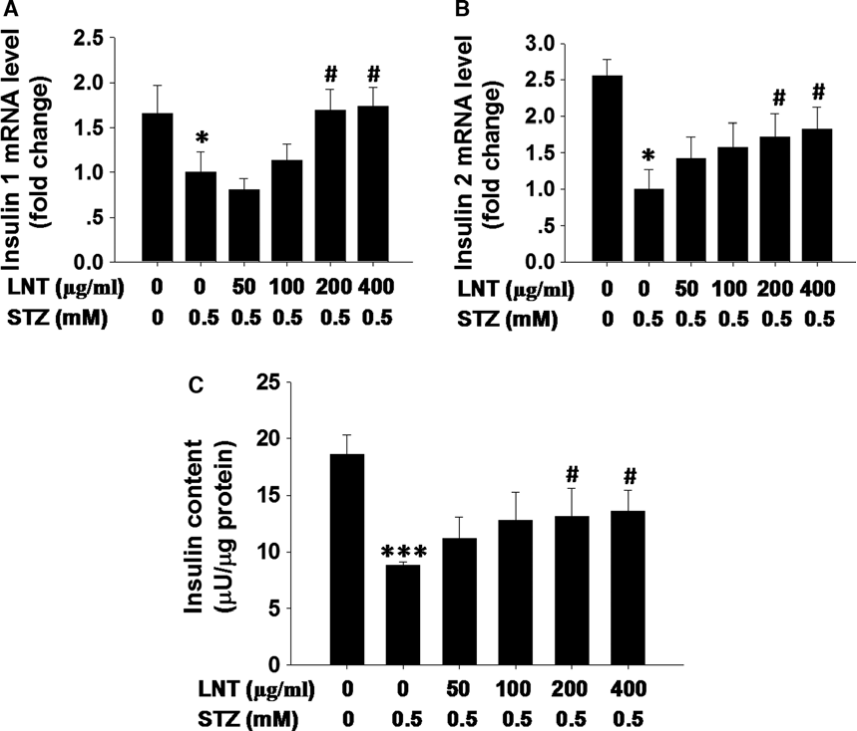
Effect of LNT on insulin synthesis in STZ‐treated INS‐1 cells. (**A** and **B**) Real‐time PCR analysis was performed to measure the level of insulin 1 (**A**) and insulin 2 (**B**) after treatment with indicated regents. (**C**) Cells pre‐treated with the indicated concentration of LNT for 30 min. were incubated with or without 0.5 mM STZ for 24 hrs. Insulin content was measured after acidified ethanol extraction. *P < 0.05, ***P < 0.001 compared to the untreated control group; ^#^ *P* < 0.05 compared to the STZ group.

Streptozotocin‐induced diabetes is characterized by extreme insulin deficiency as a result of a decrease in the number of functional β cells through a direct toxic effect of STZ on β cells 35. To detect the effect of LNT on insulin synthesis in STZ‐treated β cells, INS‐1 cells were pre‐treated with LNT for 30 min., and further treated with or without STZ for 24 hrs. As shown in Figure 5A and B, insulin 1 (Fig. 5A) and insulin 2 (Fig. 5B) transcription levels were greatly repressed in INS‐1 cells after treated with STZ, whereas incubation with 200 and 400 μg/ml LNT resulted in an significant increase in insulin transcription compared to STZ‐treated‐only controls. In addition, exposure to STZ for 24 hrs induced a marked reduction in insulin content, whereas incubation with LNT resulted in a significant improvement of insulin content (Fig. 5C). Taken together, these results demonstrated that the LNT can improve the insulin synthesis in STZ‐treated INS‐1 cells.

### LNT protects INS‐1 cells against STZ‐induced dysfunction by inhibiting the activation of NF‐κB and enhancing the expression of Pdx‐1 in INS‐1 cells

**Figure 6:**
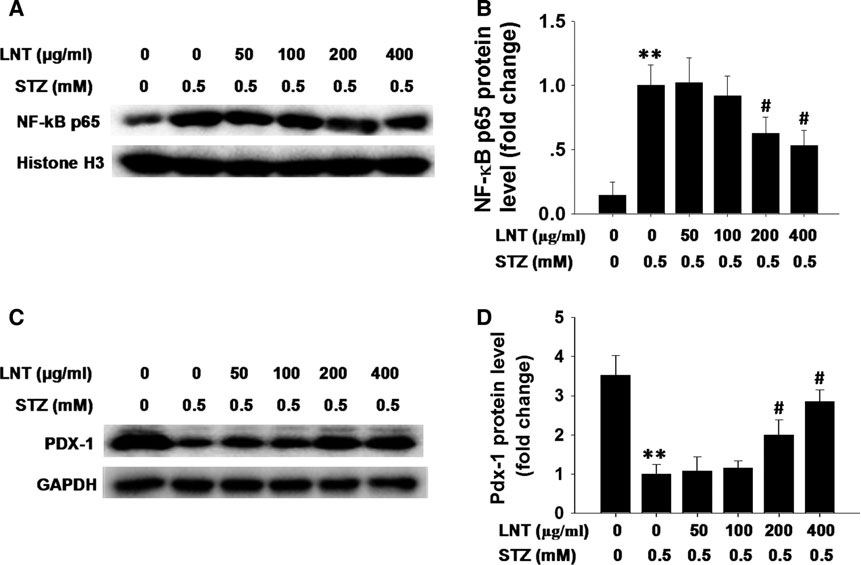
Effect of LNT on the level of nuclear NF‐κB and Pdx‐1 in STZ‐treated INS‐1 cells. (**A**) Following pre‐incubation with LNT for 30 min., the cells were treated with the indicated concentrations of LNT and STZ (0.5 mM) for 24 hrs. Nuclear NF‐κB p65 was determined by immunoblotting. (**B**) Quantification of NF‐κB p65 protein level is shown as in **A**. (**C**) INS‐1 cells were treated as in **A**, and the protein level of Pdx‐1 was determined by Western blot analysis. (**D**) Quantification of NF‐κB p65 protein level is shown as in **C**. **P < 0.01 compared with untreated control group; ^#^ *P* < 0.05 compared to the STZ group.

To further explore the possible mechanism for LNT attenuation of dysfunction in STZ‐treated INS‐1 cells, we detected the activation of NF‐κB and the level of Pdx‐1. As illustrated in Figure 6A and B, stimulation with 0.5 mM STZ strongly increased the level of nuclear NF‐κB p65 in INS‐1 cells, whereas, the level of nuclear NF‐κB p65 was significantly decreased in a dose‐dependent manner upon treatment with LNT. Importantly, in conjunction with the reduction in nuclear NF‐κB, LNT dose‐dependently prevented STZ‐induced inhibition of Pdx‐1 expression in INS‐1 cells (Fig. 6C and D), consistent with previously reported findings 36. Collectively, these results indicated that LNT suppress STZ‐induced β‐cell dysfunction by preventing NF‐κB activation, and then increasing Pdx‐1 expression.

## Discussion

Diabetes treatment and prevention requires the preservation of β cells. Mounting evidence has indicated that hyperglycaemia as well as other risk factors for diabetes can induce oxidative stress 29, 37. Because of a relatively low enzymatic antioxidative defence system, pancreatic β cells are more vulnerable to oxidative stress, which plays an important role in the development of diabetes and its complications through the induction of pancreatic β‐cell damage 29, 38. Streptozotocin, a widely used chemical to induce experimental diabetes in animals, can trigger pancreatic β‐cell damage by inducing the production of ROS 13, 39, 40. As an active ingredient obtained from the *L. edodes*, LNT has been shown to exhibit novel antioxidation bioactivity 18. In this study, we have evaluated the protective effects of LNT on STZ‐induced dysfunction in pancreatic β cells. The results demonstrate for the first time that LNT promoted cell survival and function of INS‐1 cells upon STZ toxicity.

Previous studies have shown that STZ at low concentrations (10–20 mM) induce apoptosis in INS‐1 cells, whereas, at higher concentrations (30 mM), STZ mainly induces necrosis in insulin secreting insulinoma cells 41, 42, 43. In this study, to identify the functional concentration of STZ in INS‐1 cells, a dose–response analysis was performed. A much lower concentration of STZ (0.5 mM) induced significant apoptosis in INS‐1 cells. This result might have been because of our use of a longer exposure time. Importantly, incubation with LNT effectively suppressed STZ‐induced cell apoptosis in a dose‐dependent manner, suggesting that improvement of INS‐1 cells viability by LNT might provide a new understanding of the mechanisms for its antidiabetic effects.

Numerous studies have confirmed that STZ induces reproductive toxicity mainly by inducing ROS and nitric oxide production *in vitro* and *in vivo* 40. Raza *et al*. 32 have demonstrated that STZ can induce cytotoxicity in human hepatoma cells by increasing ROS production, oxidative stress and mitochondrial dysfunction. Moreover, nitric oxide production appears to be involved in β‐cell damage, dysfunction and the pathogenesis of diabetes 44. Consistent with these studies, our study revealed that ROS generation was significantly increased in INS‐1 cells exposed to 0.5 mM STZ. In addition, the level of nitric oxide in INS‐1 cells was significantly elevated as the result of 0.5 mM STZ treatment compared to the control cells. However, ROS and nitric oxide levels in the LNT‐treated cells were reduced significantly, and this effect was concentration dependent. Excessive and sustained generation of nitric oxide derived from iNOS plays an important role in β‐cell apoptosis 45, 46. To test whether the inhibition of nitric oxide by LNT is because of the inhibition of iNOS expression, we further analysed the expression level of iNOS in LNT‐treated cells. The results demonstrated that LNT could significantly inhibit STZ‐induced iNOS protein expression. Collectively, these results suggested that the antioxidative activity might be one of the main mechanisms of the protective effect of LNT on STZ‐injured β cells.

Mitogen‐activated protein kinase signalling pathway can be triggered by a variety processes including oxidative stress, environmental stress and toxic chemical insults 33, 47, 48. In this study, to further explore whether the MAPK signalling pathway plays a role in regulating STZ‐induced apoptosis and ROS generation, we examined the activation of JNK, p38 MAPK and ERK pathways in STZ‐treated cells. As shown in Figure 3, STZ induced the activation of JNK, p38 MAPK and ERK pathways in INS‐1 cells. Interestingly, LNT was only found to dose‐dependently inhibit the activation of JNK and p38 signalling pathways in response to STZ, whereas the ERK pathway was not affected. Consistently, the level of apoptosis and ROS generation were significantly decreased in LNT‐treated cells compared to the STZ and Am co‐treatment control (Fig. 4). Together, these results suggest that the activation of the MAPK pathway in response to STZ is associated with the induction of apoptosis and ROS generation, whereas inhibition of JNK and p38 MAPK by LNT, at least in part, confers cell survival. However, further investigation of the mechanism underlying the anti‐apoptosis effects of LNT is still necessary.

Lentinan showed significant protective effects on cell viability and anti‐apoptosis in INS‐1 cells, which suggests that LNT might have a protective effect on insulin synthesis. As expected, LNT effectively suppressed STZ‐induced blockage of insulin content in a dose‐dependent manner, which demonstrated that LNT increases insulin synthesis in β cells. Previous studies have demonstrated that alloxan or STZ‐induced diabetes is linked to NF‐κB activation in pancreas in an *in vivo* model 49. Furthermore, increased generation of ROS activates the NF‐κB pathway, which is involved in the inflammation response and endoplasmic reticulum stress response of pancreatic cells 11. Interestingly, as a putative target, microarray and RT‐PCR analysis found that NF‐κB activation could decrease the level of Pdx‐1 expression and induce the β‐cell dysfunction and death 36. Thus, to further explore the possible mechanism for LNT attenuation of the dysfunction of STZ‐treated INS‐1 cells, we detected the activation of NF‐κB and the level of Pdx‐1 in LNT‐treated cells. We found that LNT suppresses STZ‐induced β‐cell dysfunction by preventing NF‐κB activation, and then increases Pdx‐1 expression. In combination with the results shown in Figure 2, these data suggest that LNT may be involved in protecting insulin‐secreting cells against STZ‐induced ROS generation, and then preventing NF‐κB activation. N^G^‐Monomethyl‐L‐arginine or L‐monomethyl N‐arginine is a well‐recognized NOS inhibitor and was chosen for many studies 50. Further investigation on the observed effect mechanism of LNT would still be necessary.

In conclusion, this study provided the first evidence that LNT protects pancreatic β cells against STZ‐induced INS‐1 cell damage, the mechanism of which might be ascribed, at least partly, to the inhibition of ROS generation, and then anti‐apoptotic effects through the prevention JNK and p38 MAPK signalling, or/and anti‐dysfunction through the prevention NF‐κB activation. These observations help elucidate the mechanisms involved in pancreatic β‐cell damage in diabetes, and provide a potential target for the treatment of diabetes with LNT. However, the findings from this *in vitro* study do not suggest that LNT would invariably exert similar effects *in vivo*. Furthermore, STZ‐induced diabetic models differ significantly from naturally occurring diabetes. In addition, LNT could also protect pancreatic β cells from other stimuli‐induced apoptosis and dysfunction, such as high levels of free fatty acids or Glucose and tumour necrosis factor‐α. Thus, more comprehensive studies are necessary to evaluate the antidiabetic effects of LNT before its clinical application.

## Conflict of interest

The authors declare that they have no conflict of interest.

## Supporting information


**Figure S1** (**A** and **B**) The survival rate of INS‐1 cells was determined in a MTT assay after treatment with the indicated concentrations of STZ (**A**) and LNT (**B**) for 24 hrs. (**C**) The Edu proliferation assay was performed 24 hrs after treatment INS‐1 cells with indicated regents. (**D**) The survival rate of INS‐1 cells was determined by MTT after treatment with indicated concentration of anisomycin (Am) for 24 hrs. *P < 0.05, **P < 0.01 and ***P < 0.001 compared to the untreated control group.
