# Supplementary figures and images for: Lentinan protects pancreatic β cells from STZ‐induced damage
Source: J Cell Mol Med. 2016 Jul 22;20(10):1803–12. doi: 10.1111/jcmm.12865 (PMC5020630; doi:10.1111/jcmm.12865)

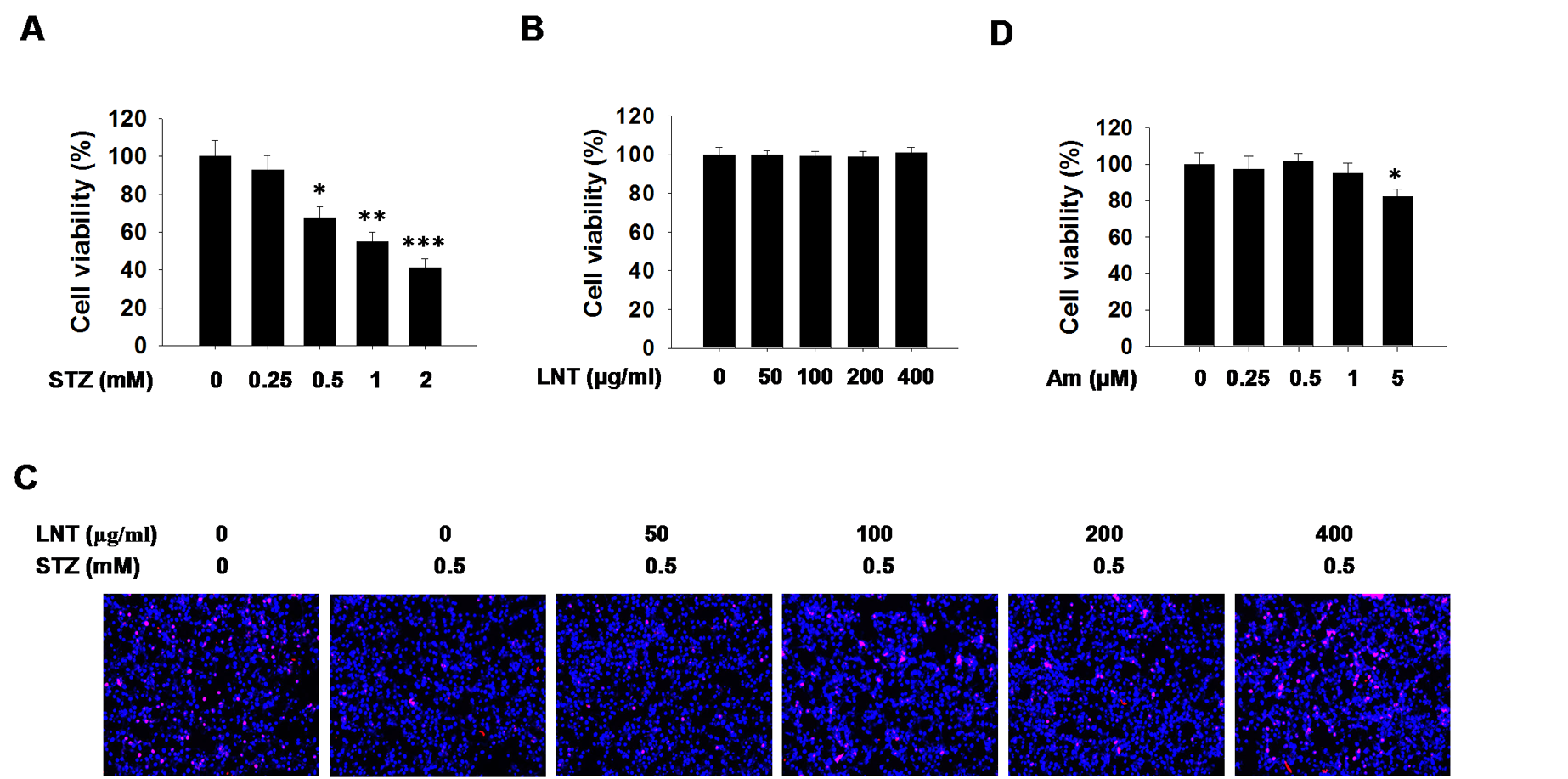

Supplement: Supplementary file 1 — Figure S1 (A and B) The survival rate of INS‐1 cells was determined in a MTT assay after treatment with the indicated concentrations of STZ (A) and LNT (B) for 24 hrs. (C) The Edu proliferation assay was performed 24 hrs after treatment INS‐1 cells with indicated regents. (D) The survival rate of INS‐1 cells was determined by MTT after treatment with indicated concentration of anisomycin (Am) for 24 hrs. *P < 0.05, **P < 0.01 and ***P < 0.001 compared to the untreated control group. [file JCMM-20-1803-s001.tif]
