# Supplementary material for: Lentinan protects pancreatic β cells from STZ‐induced damage
Source: J Cell Mol Med. 2016 Jul 22;20(10):1803–12. doi: 10.1111/jcmm.12865 (PMC5020630; doi:10.1111/jcmm.12865)
Supplement: Supplementary file 2 [file JCMM-20-1803-s002.doc]

**Supplemental data for review**

**
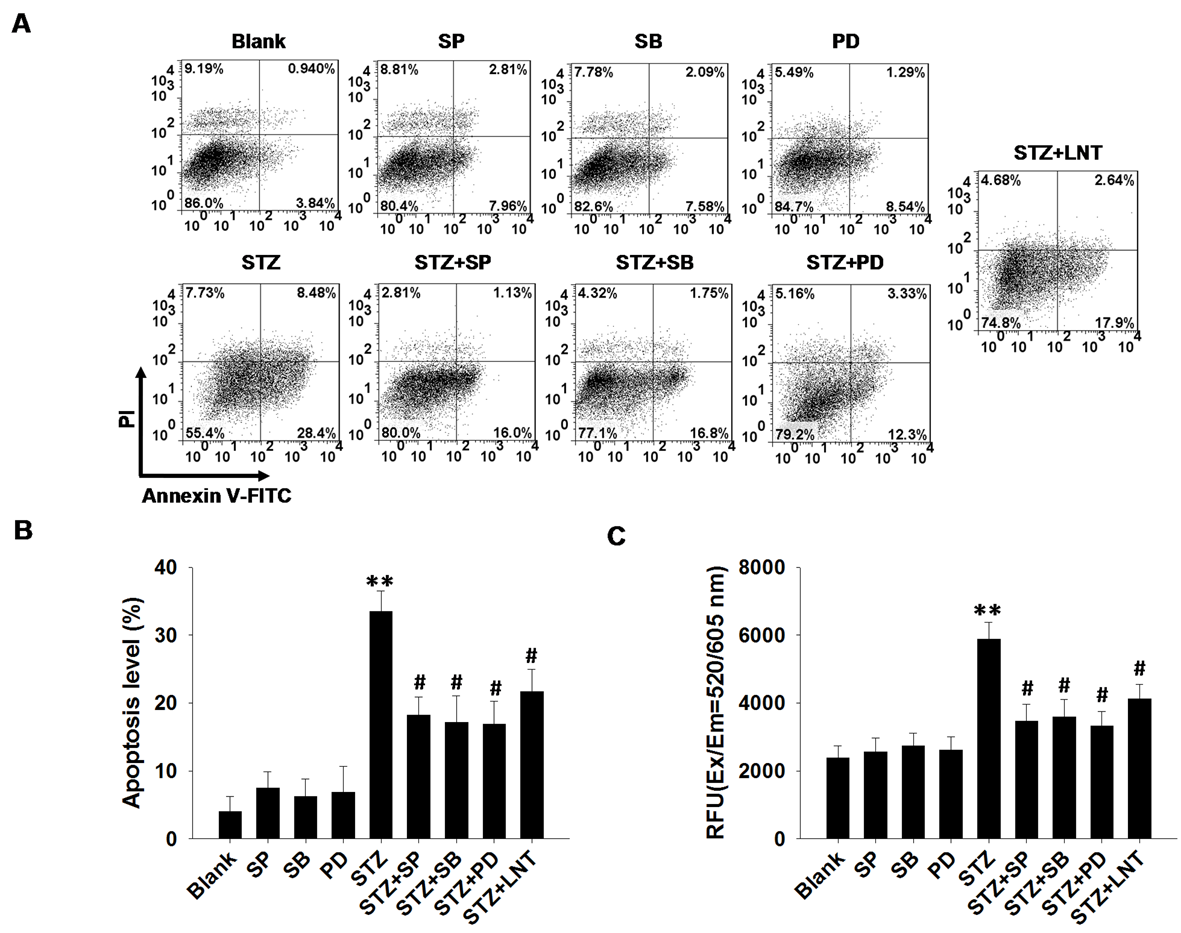
**

**Fig. S1.** (**A**) After pretreatment with LNT (200 μg/ml), SP600125 (SP, 25μM), SB203580 (SB, 20μM), or PD98059 (PD, 20μM) for 30 min, INS-1 cells were treated with STZ (0.5 mM) or/and LNT (200 μg/ml) for 24 h. Cell apoptosis was detected using flow cytometric assay with Annexin V-FITC and PI-staining. (**B**) Quantitative analysis of cellular apoptosis detected by flow cytometric measurements. (**C**) INS-1 cells were treated as in A. ROS production was analyzed using Cellular Reactive Oxygen Species Detection Assay Kit (Red Fluorescence). The fluorescence signal was monitored at Ex/Em = 520/605 nm (cut off = 590 nm) with bottom read mode. ***p* < 0.01 compared to the untreated control group; ^#^*p* < 0.05 compared to the STZ group.
